# Use of Biomarkers in Nutrition Intervention Studies of Children: A Scoping Review

**DOI:** 10.3390/nu16213584

**Published:** 2024-10-22

**Authors:** Megha P. Pratapwar, Heli J. Sheth, Anushree K. Ravi, Morgan L. Block, Kiersten A. Korber, Andrea Kepsel, Mara Leimanis-Laurens, Sarah S. Comstock

**Affiliations:** 1 Department of Food Science and Human Nutrition, Michigan State University, East Lansing, MI 48824, USA; 2 Helen DeVos Childrens Hospital, Grand Rapids, MI 49503, USA; 3 MSU Libraries, Michigan State University, East Lansing, MI 48824, USA; 4 Pediatric Critical Care Unit, Helen DeVos Children’s Hospital, 100 Michigan Street NE, Grand Rapids, MI 49503, USA; 5 Department of Pediatrics and Human Development, College of Human Medicine, Michigan State University, Life Sciences Building, 1355 Bogue Street, East Lansing, MI 48824, USA

**Keywords:** pediatrics, weight management, obesity, interventions, biomarkers

## Abstract

Obesity in youth is an increasingly prevalent public health concern worldwide. Lifestyle interventions aim to help participants establish healthy habits and reduce obesity-related disease risk by targeting physical activity and dietary habits. Most studies assess weight loss, but biomarkers may enable more rapid and comprehensive assessment of intervention success. This scoping review aims to synthesize the published literature on which biomarkers are assessed during interventions for pediatric obesity to inform future use. This review followed the Preferred Reporting Items for Systematic Reviews and Meta-Analyses (PRISMA) guidelines. A literature search of five databases conducted in February 2022 returned 1579 unique and relevant articles published between 2006 and 2021. After screening titles, abstracts, and full text, four reviewers determined that 43 studies met eligibility requirements. Quality screening was conducted, and 97.7% of papers were of fair or good quality. Of the 43 studies, 47% reported measures of adipose-related signaling molecules inclusive of adipokines, 74% included insulin-related biomarkers, 63% reported lipid-related biomarkers, 40% reported proinflammatory cytokine biomarkers, 12% reported measures of skin and/or plasma carotenoids, 40% measured blood pressure, and 21% included liver enzymes. Sixty-seven percent of studies measured biomarkers in whole blood, 40% measured biomarkers in plasma, 56% measured biomarkers in serum, and 2% measured biomarkers in urine. This work summarizes the current use of biomarkers in lifestyle intervention studies enrolling children. These biomarkers could be clinically relevant for pediatric weight management interventions.

## 1. Introduction

Obesity in children and adolescents is an increasingly prevalent public health concern in the US and globally. Data from 2017 to 2020 of the National and Nutrition Examination Survey (NHANES) indicated that the prevalence of obesity was 19.7% for US children and adolescents aged 2–19 years old [1]. Childhood obesity can lead to serious health complications later in life like type II diabetes mellitus (T2DM), metabolic syndrome, nonalcoholic fatty liver disease (NAFLD), and other chronic and endocrine disorders [2]. Modifiable factors that contribute to childhood obesity include developmental, biological, environmental, and behavioral factors [3]. Within these categories, factors like the child’s gastrointestinal microbiome, epigenetics, and intrauterine effects have recently emerged as possible contributors [3]. Obesity during childhood and adolescence is of particular concern because it is additionally linked to changes in growth and pubertal development, sleep disorders, polycystic ovarian syndrome (PCOS), psychological disorders like depression, and more [3]. Additionally, earlier-onset obesity, like during the critical developmental periods of childhood, has a higher risk of persisting throughout life [4]. Considering these factors, there is a strong need for obesity prevention strategies that are realistic and effective for a large population as well as a need for monitoring both the person-specific and program-specific success of such strategies.

A variety of prevention strategies have been employed in obesity research with children. Lifestyle interventions aim to help participants establish healthy habits and reduce obesity-related disease risk by targeting physical activity and dietary habits and are a key intervention for obesity management [3,5]. These interventions are well recognized as a beneficial choice for individuals with obesity but have only shown moderate effects on weight loss long-term [3]. More comprehensive lifestyle interventions to address pediatric obesity have become increasingly prevalent due to their multidisciplinary approach to addressing multiple factors of obesity [3]. Some proposed factors to incorporate into interventions include nutrition education and counseling, parenting skills, behavioral strategies, and pharmacological or surgical methods [6]. New intervention types like community-based, school-based, and family-based interventions have emerged from this to incorporate more of these factors related to obesity at once and help children and adolescents adopt healthier lifestyles to improve their health long-term [6]. Additionally, culinary medicine has become a rising topic among healthcare professionals to reduce the burden of chronic diseases through diet [7]. While the field of nutrition assesses how food contributes to prevention and treatment of disease, the field of culinary medicine addresses barriers to healthy eating by providing individuals with practical cooking skills along with nutrition education [7]. There are a wide variety of intervention studies whose conclusions need to be synthesized to understand their overall impact on obesity.

Though most interventions focus on weight loss as an end goal, other outcomes may be important. Lifestyle interventions targeting diet and exercise assess a broad range of outcomes, but most commonly, weight loss is the primary measure of success [8]. Participant weight loss from intensive lifestyle and behavioral interventions averages 7% to 10% of starting body weight over 52 weeks [8]. Obesity management aims to sustain weight loss of 10% or more in order to have the greatest improvements in obesity-related complications [8]. Aside from body mass index (BMI), many studies investigating lifestyle interventions report other outcomes which could potentially act as improved biomarkers of success. Specifically, some studies focus on specific biomarkers related to chronic disease risk. Biomarkers related to oxidative stress, insulin resistance, and inflammation are often studied because they are known contributors to the development of diabetes and NAFLD, common downstream disease outcomes for those with obesity [9]. The nine categories of biomarkers (adipose-related signaling molecules; anthropometry; insulin-related; lipids; proinflammatory cytokines; carotenoids; blood pressure; liver enzymes; other biomarkers) reported herein were decided upon after completing the screening of included manuscripts and based on the list of biomarkers that emerged from that process. 

These biomarkers are important because they can help distinguish the populations of obese individuals who are metabolically healthy versus those who are not, and, therefore, quantify the biological efficacy of lifestyle interventions for obesity [9]. The extracted data are reported as frequencies, which allows for other researchers to know which biomarker measurements will be most commonly available for comparison with their own work in this field. 

The aim of this scoping review is to synthesize the published literature on which biomarkers are assessed during interventions for pediatric obesity over a 15-year time period (2006–2021) and to determine the most frequently measured biomarkers in intervention studies with children and adolescents. Results from this review of the literature can help direct biomarker use in future intervention trials in this population.

## 2. Materials and Methods

A research librarian (A.K.) designed and conducted a search strategy in the following databases: PubMed/MEDLINE, Embase (Elsevier), Scopus, Web of Science (limited to Biological Abstracts, CAB Abstracts, Food Science and Technology Abstracts), and ProQuest Theses and Dissertations Global. The literature search was conducted on 7 February 2022, using a combination of keywords and subject terms for gut microbiome, adolescents at risk for obesity, and dietary behavior. Controlled vocabulary, such as Medical Subject Headings (MeSH) and Emtree, were used where appropriate. No limits were applied for publication date or language, but keywords were limited to title and abstract fields. Complete search strategies for each database are included in the Supplemental Material.

The literature search produced 2073 results across 5 databases. Unduplicated citations from each database were as follows: PubMed, 363; Embase, 775; Scopus, 57; Web of Science, 857; and ProQuest Theses and Dissertations, 21. Citations were imported into an EndNote library, where duplicates were removed (365) and an initial screening was performed to remove incomplete or irrelevant records (129). To be included, studies needed to include children between 8 and 13 years of age, measure biomarkers, and include lifestyle intervention. Other inclusion criteria included publication in the last 15 years and being written in English. Exclusion criteria included studies conducted on non-humans, adults, or children with underlying conditions (with the exception of overweight or obesity), and studies which reported only change in weight, not other biomarkers. 

**Figure 1:**
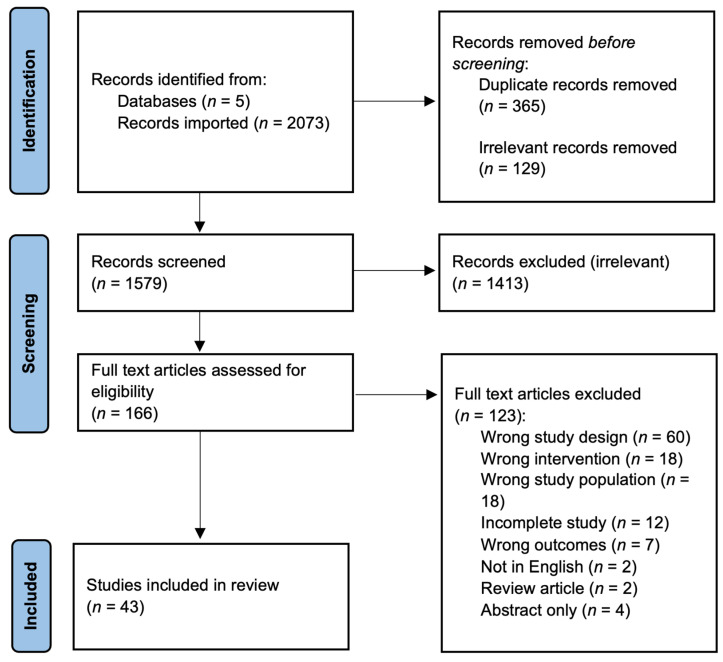
Preferred Reporting Items for Systematic reviews and Meta-Analyses (PRISMA) flow diagram of abstract and full text screening [10].

The remaining 1579 citations were uploaded to Covidence (Melbourne, Australia) for screening. Title and abstract screening was completed by two independent reviewers for each study, and consensus was reached by a third independent reviewer. The remaining 166 full-text articles were then each assessed by two independent reviewers for further eligibility based on inclusion criteria, and a third independent reviewer determined consensus (Figure 1). The screening process followed the guidelines of the Preferred Reporting Items for Systematic reviews and Meta-Analyses (PRISMA), which is displayed in the PRISMA flow diagram (Figure 1). From this, 43 studies met the eligibility requirements and were used for analysis in the scoping review. Data from these 43 studies were extracted using the Qualtrics XM survey tool (Provo, UT, USA). Each study was extracted by two reviewers, and any discrepancies were settled by a third reviewer.

All 43 articles included in this review underwent quality screening through use of a revised Downs and Black scale. [11] The complete Downs and Black scale consists of a 27-methodological-question checklist [11]; however, 8 of these questions were omitted as they were not applicable to the studies included in this review. A modified version of the Downs and Black scale has also been utilized in other review articles [12,13,14]. Using this scale, the quality of a study is assessed based on reporting, external validity, internal validity, and statistical power [11]. Studies were categorized as being of “poor quality”, “fair quality”, or “good quality”. To categorize a study, numerical values were first assigned for each response to questions in the checklist, with 1 for “yes”, 0 for “no”, and 0 for “unable to determine”. After each article was assessed for quality and responses were recorded, the resulting numerical values were summed to provide a final numerical score for each article. The final numerical value score translated to a quality category based on the following parameters: ≤8 is “poor quality”, >8 to ≤12 is “fair quality”, and >12 is “good quality”. Of the 43 articles included in this review, 1 (2.3%) was classified as poor quality, 14 (32.6%) were classified as fair quality, and 28 (65.1%) were classified as good quality (Supplemental Table S1). For the set of articles included in this review, the average numerical modified Downs and Black quality score was 13 with a median of 14, a min of 7, and a max of 19. The study classified as ‘poor quality’ lacked information on reporting of confounders, main findings of the study, study population, statistical analysis, and statistical power, therefore affecting the validity and reliability of the study. However, we included this study in the scoping review because the focus of the review is the biomarkers measured rather than the values or changes in those biomarkers with treatment.

## 3. Results

### 3.1. Overall Set of Publications Included

#### Study Characteristics

This review examined 43 studies, including studies that were conducted in various countries. Twenty-three percent of papers included participants from solely the United States. A total of 9% of studies included participants from Mexico, 12% from Spain, 9% from Poland, 9% from Germany, and 5% from Denmark. Countries only represented in a single study included China, Italy, Thailand, Korea, Austria, Portugal, Belgium, Hungary, Brazil, Iran, and Greece. Moreover, 2 of the 43 studies are multinational, with one study having participants from US and Germany, and the other having participants from multiple European countries.

**Figure 2:**
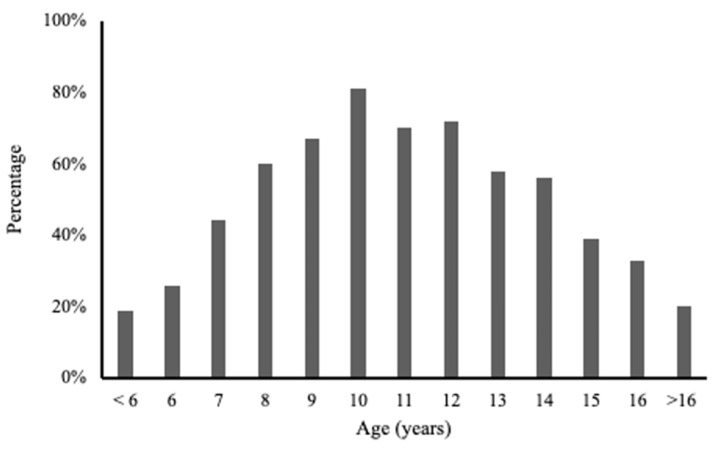
Percent of the 43 papers containing participants in each age group.

The length of intervention across the 43 studies varied widely. The shortest intervention time was 2 weeks, and the longest was 2 years. Sixty-two percent of the 43 papers did not include the specific start and end dates of the study but did report intervention duration. The most common ages included in the papers were ages 8 to 14 years, but participants ranged in age from 2 years to 19 years (Figure 2).

Along with dietary interventions, many studies used additional intervention components. Physical interventions such as exercise stations, participation in sports, moderate exercise, high-intensity interval exercise and resistance training were also used with dietary intervention in 88% of studies. Other interventions such as outpatient group-based sessions, group classes, coaching, and psychological family therapy were used with dietary intervention in 49% of studies.

The interventions in the studies were conducted in a variety of locations. A total of 16% of studies used school-based intervention, 16% used community-based intervention, 58% used family-based intervention, and 58% used outpatient-based intervention. Many studies used more than one intervention location.

The definition used for obesity/overweight was given in all but five studies. The most common method used to define obesity was participant BMI in 83% of studies. Only 5% of papers used adiposity to define obesity/overweight status.

In the 43 studies, distinct biomarkers were measured from a variety of biospecimens. A total of 67% of studies measured biomarkers in whole blood, 40% measured biomarkers in plasma, 56% measured biomarkers in serum, and 2% measured biomarkers in urine. No studies included in this review measured biomarkers in stool samples. 

The categories of biomarkers were determined after screening of manuscripts and compiling and sorting the resulting list of biomarkers. Biomarkers that appeared in a majority of the papers received their own category whereas biomarkers that were mentioned once or rarely were included in the “other” category.

Of the 43 studies, 47% of the studies reported measures of adipose-related signaling molecules including adipokines, 74% included glucose- or insulin-related biomarkers, 63% reported lipid-related biomarkers, 40% reported proinflammatory cytokine biomarkers, 12% reported measures of skin carotenoids and/or plasma carotenoids, 40% measured blood pressure, and 21% included liver enzymes. Within the 43 studies, there were other biomarkers measured that did not fall into the biomarker categories previously listed. These other biomarkers are listed in the table below (Table 1).

### 3.2. Biomarker Specific Publications

#### 3.2.1. Adipose-Related Signaling Molecules

Of the 43 papers included in this review, 20 (47%) included measurements of adipose related signaling molecules (Table 2). Leptin and adiponectin were most commonly included. In fact, from the studies examining adipose-related signaling molecules, 95% of the papers included leptin, 75% included adiponectin, and 10% included resistin. The most common anthropometric measurement measured concurrently with adipokines was BMI/body mass (45% of studies). Both papers that examined resistin levels in plasma found levels to be significantly lower after the intervention. Fifty-five percent of the papers examining adiponectin in blood samples found that levels increased after the intervention. These adipose-related signaling molecules were the most commonly measured in children regardless of participant age.

#### 3.2.2. Blood Pressure

Seventeen (40%) of the included papers reported participant blood pressure (Table 3). All 17 examined both diastolic and systolic blood pressure in relation to the intervention. Fifty-nine percent of the papers demonstrated that the intervention resulted in a reduction in blood pressure. Only one paper [28] did not report an association between the treatment, the Mediterranean diet, and a decrease in blood pressure. The researchers in [28] hypothesize that this is because the participants exhibited both sex- and age-appropriate values in their baseline measurements. Meanwhile, the researchers in [26] observed a decrease in blood pressure during the intervention but then observed increased levels at the one-year follow-up visit. In [50], the researchers identified an increase in diastolic blood pressure after the intervention. 

#### 3.2.3. Liver-Associated Biomarkers

A total of 9 of the 43 papers (21%) examined reported information regarding hepatic-associated biomarkers (Table 4). Typically, liver enzymes were measured in blood/serum. Primarily, these papers investigated aspartate transaminase (AST) and alanine transaminase (ALT), among other enzymes. AST and ALT are liver enzymes commonly measured in clinical settings to indicate appropriate functioning of the liver and become variably elevated during liver injury, ultimately aiding in classifying underlying liver pathology. Fifty-six percent of the nine papers referenced liver enzymes, AST and ALT. Only four of the nine studies (44%) examining liver enzymes reported significant changes after the intervention in a variety of biomarkers in each study. Pedrosa [35]) was the only study to examine and report a positive association between the intervention and Gamma-glutamyl Transferase (GGT), an enzyme indicative of bile duct damage when critically elevated. Liver enzymes were positively associated with short-chain fatty acids in blood [22]. Both papers that included assessments of fibrinogen in blood found that fibrinogen was higher in obese individuals and correlated with a reduction after a lifestyle-based intervention [16,57]). 

#### 3.2.4. Blood Glucose and Insulin-Related Biomarkers

Of the papers included in this review, 74% included assessments of blood glucose and insulin-related biomarkers (Table 5). Among those 32 papers, glucose, insulin, and insulin resistance (HOMA-IR) were most commonly measured and reported. Less commonly, fasting insulin, 2 h glucose, and hemoglobin A1c (HbA1c) were measured and reported. Rarely, the quantitative insulin sensitivity check index (QUICKI) and acute insulin response (AIR) were measured and reported. Twenty-nine papers (91%) reported plasma glucose levels. Of those, 2 (7%) reported plasma glucose, 27 (93%) reported fasting plasma glucose, and 5 (17%) reported plasma glucose with a 2 h glucose test. Moreover, 5 (16%) of the 32 papers used two or more tests to assess insulin levels, aside from plasma insulin levels. Additionally, 78% of papers reported insulin levels, with 21 total papers reporting plasma insulin, while 15 specifically reported fasting plasma insulin levels. Moreover, 66% of the papers examined HOMA-IR, while 19% examined HbA1c.

#### 3.2.5. Lipid Biomarkers

In the 43 papers included in this study, 27 (63%) reported lipid biomarkers (Table 6). These were measured in fasting whole blood samples in 89% of studies, with one study measuring non-fasting and two studies making no mention of fasting status. Triglycerides (TG), total cholesterol, high-density lipoprotein cholesterol (HDL), low-density lipoprotein cholesterol (LDL), Apolipoprotein B (cholesterol), and Apolipoprotein A-I (cholesterol) were the most common lipid biomarkers. Less commonly included lipid biomarkers were very low-density lipoprotein (VLDL), lipid accumulation profile (LAP), LDL/HDL ratio, non-HDL, and TG/HDL ratio. Out of the 27 papers including lipids, 100% of them measured triglycerides and total cholesterol. Moreover, 96% of the papers measured HDL, and 93% included LDL as a lipid biomarker. Eleven percent of the 27 papers included Apolipoprotein B and Apolipoprotein A-I as a lipid biomarker.

#### 3.2.6. Proinflammatory Cytokine Biomarkers

Of the 43 papers included in this study, 17 (40%) included measurement of proinflammatory cytokine biomarkers (Table 7). The most frequently measured biomarkers were TNF-α (35%), IL-6 (47%), and C-reactive protein (94%). Of the papers including a measurement of CRP, 23.5% measured CRP and 71% specifically measured hsCRP (high-sensitivity C-reactive protein). Biomarkers measured only once in all 43 studies include serum amyloid A (SAA), MCP-1, MPO, IL-8, IL-1, IL-1 receptor antagonist (IL-1ra), IL-1β, IL-10, and soluble CD163.

#### 3.2.7. Carotenoid Biomarkers

Of the papers included in this review, five papers (12%) included measurement of carotenoids (Table 8). Of these five papers measuring carotenoids, four papers included measurement of skin carotenoids, and one paper included measurement of plasma carotenoids. Skin carotenoids were measured using resonance Raman spectroscopy [15,41] or reflectance spectroscopy [27,31]. The plasma carotenoids included in the study by Matthan were lutein, zeaxanthin, β-cryptoxanthin, β-carotene, and lycopene.

## 4. Discussion

This scoping review investigated the results of 43 lifestyle interventions that addressed one or more biomarkers of pediatric obesity in the categories of adipose-related signaling molecules, insulin-related biomarkers, lipid biomarkers, proinflammatory cytokines, carotenoids, blood pressure, liver enzymes, and other biomarkers. The interventions assessed in this review exemplify the increasing use of diverse biomarkers to assess the efficacy of interventions for pediatric obesity. The most commonly measured feature was anthropometry, which was measured in 91% of studies. After anthropometry, 74% of studies measured glucose- and insulin-related biomarkers, most commonly plasma glucose, plasma insulin, and HOMA-IR. Glucose and insulin biomarkers are key indicators of T2DM risk, and their use is crucial in identifying and addressing prediabetes in youth populations through lifestyle modifications [58]. Sixty-three percent measured lipid related biomarkers, most commonly triglycerides, TC, HDL, and LDL. The association between obesity and dyslipidemia, specifically high amounts of triglyceride-rich lipoproteins like LDL and low amounts of HDL, is well studied and supports lipid biomarkers as being a reliable measurement of metabolic status [59]. Biomarkers like plasma glucose and triglycerides are also analyzed in a standard basal metabolic panel or lipid panel of blood, making them convenient variables to collect. Adipose-related signaling molecules (47%), proinflammatory cytokines (40%), and blood pressure (40%) were the next most often measured groups of biomarkers. Less frequently measured were liver enzymes (21%). It is also notable how few studies measured biomarkers through non-invasive methods like skin carotenoids, urine, and stool at 12%, 2%, and 0% of studies, respectively. These methods have the advantage of being easier to collect than blood measures, since children and their families may be opposed to having their blood drawn multiple times. These results provide insight into the variety of biomarkers that are commonly assessed in pediatric obesity interventions.

Of the 43 studies included in our review, 1 study assessed the patterns in biomarker changes both immediately post-intervention and during a follow-up period to understand whether the interventions had lasting impacts. High blood pressure in childhood and adolescence is a common comorbidity of obesity and a major health risk for heart disease and strokes as adults [60]. Fifty-nine percent of the studies that measured blood pressure found a significant decrease immediately after the intervention. Two of those ten studies also found that blood pressure returned to baseline levels at the 1-year timepoint assessment. These results suggest that blood pressure is a moderately effective indicator of a successful lifestyle intervention, but blood pressure may not be an effective biomarker of lifestyle changes long-term post-intervention. However, more research should be conducted on blood pressure as a biomarker of pediatric obesity due to its importance for cardiovascular health and non-invasive measurement. Similarly, only a moderate number of studies, 44%, that measured liver enzymes found significant changes in their levels post-intervention. Additionally, the study by Kazankov [26]) saw a significant decrease in ALT after the 10-week intervention, but the levels returned to the baseline values at the 12-month measurement. Overall, AST and ALT levels saw fewer changes with shorter intervention lengths. Elevated liver enzymes are a key indicator of liver damage and an important tool in screening for pediatric NAFLD, which is a common complication of obesity [61]. While liver enzymes are important blood biomarkers of chronic diseases, more research should be conducted on their use as a biomarker of interventions for pediatric obesity. Specifically, whether liver enzyme levels are responsive to lifestyle changes in shorter pediatric interventions is currently unknown.

Adipose-related signaling molecules, which include leptin, adiponectin, resistin, and ghrelin, are a group of biomarkers whose use in intervention studies is growing. Forty-two percent of studies included in this review measured at least one of these molecules. Leptin and adiponectin are frequently measured due to their properties as proinflammatory and anti-inflammatory cytokines, respectively [62,63]. Specifically, leptin was measured in 17 of the 18 studies and their results showed no conclusive relation to BMI. This result is interesting, since it challenges the common assertion that serum leptin concentrations are positively correlated with percentage body fat [62]. However, 54% of the studies in our review that measured adiponectin found an increase in their levels post-intervention. These results are relatively consistent with previous research that adiponectin is inversely associated with metabolic disease risk, supporting its classification as an anti-inflammatory adipokine [62]. Two studies measured resistin levels and found a significant decrease after completing the intervention. While leptin and adiponectin as individual biomarkers in our current review do not show consistent associations, new research indicates that the ratio of leptin to adiponectin (L/A) has greater accuracy as a biomarker for insulin resistance in adolescents [63]. Only one study in our current review discussed the L/A ratio as a biomarker; however, they did not report the exact values, only that the L/A was lower after the 12-month intervention [52]. Studies that have measured both leptin and adiponectin may benefit from calculating the additional L/A variable to increase their accuracy of intervention assessment. Further research should be conducted to confirm the consistency of leptin, adiponectin, and resistin’s associations with BMI and metabolic status, as well as to confirm the efficacy of the L/A ratio.

Proinflammatory cytokines are useful biomarkers to assess inflammation associated with chronic diseases. We found 17 studies in our review that measured 12 different proinflammatory cytokine biomarkers, the most frequently measured include C-reactive protein (94%), IL-6 (47%), and TNF-α (35%). CRP is an acute-phase protein that is produced by the liver in a state of infection, inflammation, or malignancies [64]. The production of CRP is regulated by cytokines like IL-6 [64]. A total of 7 of the 43 studies in our review measured both CRP and IL-6 levels. High-sensitivity CRP measurements became more common for their ability to detect lower levels of CRP that may indicate cardiovascular risk. Recently, standard CRP assays have become more sensitive and are preferred by researchers due to their lower cost. Both CRP and hsCRP, in coordination with other biomarkers like lipid profile, can aid in determining low, medium, and high cardiovascular risk [64]. However, since CRP is a nonspecific biomarker, many different sources of inflammation may influence levels and confound the direct association with cardiovascular risk [64]. Therefore, CRP and hsCRP can be effective biomarkers of interventions but should be carefully analyzed against other variables as well. Another commonly measured biomarker is tumor necrosis factor alpha (TNF-α), which is a cytokine released by adipocytes in response to chronic inflammation [65]. A study by Alzamil [65] found that TNF-α was significantly higher in individuals with obesity and T2DM, and that TNF-α was positively correlated with HOMA-IR and HbA1c [65]. However, the current literature is inconclusive about the relationship between TNF-α and obesity or T2DM. Accordingly, only two of the six studies that measured TNF-α found a significant decrease in levels post-intervention [22,55]. Further research should be conducted into TNF-α as a biomarker for inflammation related to obesity or T2DM.

There are many strengths and limitations of this review that should be acknowledged. One strength is that we used broad search criteria which included diverse terms and multiple databases including PubMed, Embase, Scopus, Web of Science, and ProQuest Theses and Dissertations. The results of these studies are relatively easily comparable since only lifestyle intervention studies were included. We mitigated discrepancies in article screening by having two reviewers at each step and a third reviewer to resolve conflicts. While this search was thorough, it is possible that some relevant studies may have been missed. Additionally, any studies published between the search date and the time of publication could not be included in the analysis. Another limitation of this study was the heterogeneity of study population ages. Studies were included if the participant age range included any 8–13 year old individuals; however, this allowed for participants in the included studies to be anywhere from 2 to 19 years old. Therefore, some studies were compared despite having participant age ranges that did not overlap. This could be significant since children and adolescents have differences in growth, sexual maturation, and neurocognitive development, which could impact biomarker responses to interventions [66].

The insights from this review can aid in directing future studies on lifestyle interventions to address pediatric obesity. First, this review investigated a wide variety of biomarkers found in obesity intervention studies. There are opportunities to enrich future interventions by including novel biomarkers related to obesity that can be easily measured. The gut microbiome has the potential to be a powerful biomarker to assess lifestyle changes because its composition is sensitive to changes in environmental factors like diet [67]. In addition to measures of overall microbiome composition like diversity and richness, specific taxonomic differences have been associated with obesity and health status [68]. For example, the Firmicutes-to-Bacteroidetes ratio of bacterial phyla is often studied as a microbial signature of obesity in the gut microbiome, but conflicting claims about its validity indicate a need to further research on this particular biomarker [69,70]. Skin carotenoids are also good biomarkers for children and adolescents since their measurement is non-invasive [71]. Additionally, skin carotenoid levels are associated with carotenoid intake through fruits and vegetables, which can be used to assess compliance with dietary interventions [71]. While there were four studies in this review which investigated skin carotenoids, additional studies are needed to broaden our understanding of skin carotenoids as a biomarker of dietary change in pediatric obesity intervention studies. Future studies may benefit from continuing to expand biomarker use past anthropometry (measured in 91%) to include biomarkers from any of these categories. Researchers should continue to use glucose/insulin biomarkers and lipid biomarkers in intervention studies due to their close association with obesity and T2DM. They may also benefit from including novel biomarkers like the L/A ratio that add minimal extra study costs and have shown associations with obesity. Though currently under-utilized, skin carotenoids, urine biomarkers, and gut microbiome composition are useful non-invasive biomarkers that can aid in assessing the effectiveness of lifestyle changes. The main focus of this review was to assess the frequency of use of various biomarkers in intervention studies with children and adolescents. Additionally, we noticed increasing measurement of carotenoids after 2016. After 2011, assessment of proinflammatory biomarkers became more common. Blood pressure and liver enzyme measurement became less common over time.

Lastly, little of the published literature reports the use of biomarkers as motivation for health behavior change. A preliminary study from 2002 found that biomarkers could be a useful treatment aid in intervention studies by conveying personal risk to participants [72]. The biomarkers investigated in our current review as well as those previously mentioned in this paragraph would provide an effective basis to further study the motivational aspect of biomarker measurement and return-of-results for compliance to and retention in lifestyle intervention to address pediatric obesity.

## 5. Conclusions

Lifestyle interventions are crucial in pediatric obesity management to reduce present and future risk of chronic diseases. This scoping review evaluated 43 articles, published between 2006 and 2021, and their use of various intervention styles and biomarker measurements to assess the biomarkers measured in lifestyle interventions for obesity in pediatric populations. The most frequently measured biomarkers include glucose- and insulin-related biomarkers (74%), lipid biomarkers (63%), adipose-related signaling molecules (47%), proinflammatory cytokines (40%), and blood pressure (40%). Less commonly measured biomarker categories include liver enzymes (21%) and carotenoids (12%). Further research should be conducted to enhance our understanding of these biomarkers to address our youth population’s worsening metabolic health.

## Figures and Tables

**Table 1:** Biomarker measurements reported in each of the 43 papers included in this review.

Lead Author	Year Published	PMID	Adipose-Related Signaling Molecules ^a^	Anthropometry	Insulin-Related	Lipids	Proinflammatory Cytokines	Carotenoids ^b^	Blood Pressure	Liver Enzymes	Other Biomarkers
Pratt [15]	2021	34209574		X				X			
Huang [16]	2010	21198775		X	X	X			X	X	Heartrate, sE, sICAM-1, sVCAM-1, PAI-1
Liao [17]	2021	3207257	X	X	X	X					
Morell-Azanza [18]	2020	3200733		X	X						Telomere length
Thomsen [19]	2021	3418024	X	X	X		X		X		Midregional proatrial natriuretic peptide
Cambuli [20]	2008	1849275	X	X	X	X					
Gajewska [21]	2013	2377179	X	X							BMC, BMD, BALP, CTX-I, Leptin receptor
Matthan [22]	2020	3192208	X	X	X	X	X	X	X	X	Fat-soluble Vitamins=vitamins (A, D, E, and K), desaturase enzyme indexes (SCD1, SCD2, D6D, D5D) Fatty acids (SFA, MUFA, PUFA n-6s, PUFA n-3s, Trans), E-selectin, pg/mL P-selectin, pg/mL sICAM, ng/mL, Thrombomodulin
Santiprabhob [23]	2018	29858907	X	X	X	X	X				GPx, SOD, pMDA, ba-PWV
Arauz Boudreau [24]	2013	23415190		X	X	X	X			X	
Vilchis-Gil [25]	2018	29402762		X	X	X					
Kazankov [26]	2015	25073966		X	X	X	X		X	X	
Hopkins [27]	2017	N/A						X			
Velazquez-Lopez [28]	2014	24997634		X	X	X			X		
Reinehr [29]	2010	20065970	X	X	X						Fasting serum OC
Reinehr [30]	2009	19442975		X	X	X			X		
Juarez [31]	2019	N/A						X			
Sohn [32]	2022	35050149		X							Serum metabolites and BCAA
Mayerhofer [33]	2020	32154197	X	X	X		X				Absolute neutrophil count
Rosenbaum [34]	2007	17090635		X	X	X	X				ACRP30, GDI
Pedrosa [35]	2011	21107585		X	X	X	X		X	X	
Gajewska [36]	2009	20081271	X		X	X					sOB-R
Fernández-Ruiz [37]	2021	33517762		X	X	X					
Moleres [38]	2013	23475851	X	X	X	X	X		X		genomic DNA, DNA methylation
Gajewska [39]	2011	22006484	X	X							
Mårild [40]	2015	26707015		X	X	X	X		X		metabolic score
Beccarelli [41]	2016	N/A		X				X			desaturase enzyme activity (not included the article)
Verbiest [42]	2021	34333112		X			X				
Macknin [43]	2015	25684089		X	X	X	X		X	X	MPO
Leal-Witt [44]	2018	30204940		X	X	X			X	X	Urine metabolites (NMR), creatinine levels
Reinehr [45]	2006	16702998	X	X	X						Pancreatic polypeptide
Koncsos [46]	2011	22081619	X	X	X	X	X		X	X	Hemoglobin, hematocrit, white blood cell count, sedimentation rate, urea, creatinine, creatine kinase, PON1 paraoxonase and aryl-esterase activity, sE, ADMA
Fernandes [47]	2020	32621732	_	X	X	X					basal metabolic rate, PYY
Schwandt [48]	2011	21996754		X	X	X			X		
Reinehr [49]	2009	19922035	X	X	X	X			X		
Karampatsou [50]	2021	33924457	X	X	X	X	X		X		TSH, T3, Free T4, anti-TPO, anti-TG, ferritin, 25-OH Vitamin D, urea, creatinine, irisin, FGF-21
Gajewska [51]	2018	29192796	X	X	X	X					BMC, BMD, BALP, CTX-I, OC, CTX-I/OC, Glu-OC, Gla-OC, sOB-R, leptin/sOB-R
McFarlin [52]	2013	22458649	X	X							
Hajihashemi [53]	2014	24478050	X	X			X				sICAM-1, sVCAM-1
Pablos [54]	2018	29136476		X	X	X			X		
Izadpanah [55]	2012	22713506	X	X	X	X	X		X		PAI–1, amylin
Ojeda-Rodríguez [56]	2021	32871095		X							Telomere length
Kahhan [57]	2021	33650888	X	X	X	X	X			X	

“X” indicates measurement of biomarker in selected study. a = “adipose-related signaling molecules” includes adipokines. b = “carotenoids” includes skin and plasma carotenoids. Abbreviations: sE, selectin E; sICAM-1, soluble intercellular adhesion molecule 1; sVCAM-1, soluble vascular adhesion molecule1; PAI-1, plasminogen activator inhibitor-1; BMC, total bone mineral content; BMD; total bone mineral density; BALP, bone alkaline phosphatase; CTX-I, C-terminal telopeptide of type I collagen; GPx, glutathione peroxidase; SOD, superoxide dismutase; pMDA, plasma malondialdehyde; ba-PWV, brachial-ankle pulse wave velocity; BCAA, branched chain amino acids; ACRP30, adipocyte complement-related protein of 30 kDa; MPO, myeloperoxidase; ADMA, asymmetric dimethylarginine; TSH, thyroid-stimulating hormone; T3, triiodothyronine; Free T4, free thyroxine; anti-TPO, anti-thyroid peroxidase; anti-TG, antithyroglobulin antibody; 25-OH Vitamin D, total 25-hydroxyvitamin D; OC, osteocalcin; Glu-OC, Undercarboxylated Osteocalcin; Gla-OC, Gla-type Osteocalcin; sOB-R, soluble leptin receptor; PYY, peptide YY; GDI, glucose disposal index.

**Table 2:** Biomarkers reported in any papers examining adipose-related signaling molecules.

Lead Author	Year Published	Leptin	Adiponectin	Other Variables
Liao [17]	2021	X		Ghrelin
Thomsen [19]	2021	X	X	
Cambuli [20]	2008	X	X	
Gajewska [21]	2013	X	X	
Matthan [22]	2020	X	X	
Santiprabhob [23]	2018	X		HMW adiponectin
Reinehr [29]	2010	X	X	
Mayerhofer [33]	2020	X	X	
Gajewska [36]	2009	X		
Moleres [38]	2013	X	X	
Gajewska [39]	2011		X	
Reinehr [45]	2006	X		
Koncsos [46]	2011	X	X	
Reinehr [49]	2009	X		
Karampatsou [50]	2021	X	X	
Gajewska [51]	2018	X	X	
McFarlin [52]	2013	X	X	Resistin
Hajihashemi [53]	2014	X		
Izadpanah [55]	2012	X	X	Resistin
Kahhan [57]	2021		X	HMW adiponectin, HMW/total adiponectin ratio

“X” indicates measurement of biomarker in selected study. Abbreviations: HMW adiponectin, high-molecular-weight adiponectin.

**Table 3:** Biomarkers reported in any papers examining blood pressure.

Lead Author	Year Published	Systolic	Diastolic
Matthan [22]	2020	X	X
Kazankov [26]	2015	X	X
Velazquez-Lopez [28]	2014	X	X
Marild [40]	2015	X	X
Schwandt [48]	2011	X	X
Reinehr [30]	2009	X	X
Pablos [54]	2018	X	X
Huang [16]	2011	X	X
Thomsen [19]	2021	X	X
Reinehr [49]	2009	X	X
Pedrosa [35]	2011	X	X
Moleres [38]	2013	X	X
Macknin [43]	2015	X	X
Leal-Witt [44]	2018	X	X
Koncsos [46]	2011	X	X
Karampatsou [50]	2021	X	X
Izadpanah [55]	2012	X	X

“X” indicates measurement of biomarker in selected study.

**Table 4:** Biomarkers reported in any papers examining liver enzymes.

Lead Author	Year Published	AST	ALT	ALP	GGT	PON1	Fibrinogen
Matthan [22]	2020	X	X	X			
Arauz Boudreau [24]	2013	X	X				
Pedrosa [35]	2011	X	X		X		
Leal-Witt [44]	2018	X	X				
Koncsos [46]	2011					X	
Kahhan [57]	2021						X
Huang [16]	2011						X
Kazankov [26]	2015		X				
Macknin [43]	2015	X	X				

“X” indicates measurement of biomarker in selected study. Abbreviations: aspartate transaminase (AST), alanine transaminase (ALT), alkaline phosphatase (ALP), Gamma-glutamyl Transferase (GGT), and paraoxonase-1 (PON1).

**Table 5:** Biomarkers measured in papers containing blood glucose and insulin-related biomarkers.

Lead Author	Year Published	Glc	Fasting Glc	2-Hour Glc Test	Insulin	Fasting Insulin	HOMA-IR	HbA1c	Others
Huang [16]	2011		X		X		X		
Liao [17]	2021		X						
Morell-Azanza [18]	2020		X		X		X		
Thomsen [19]	2021		X		X		X		
Cambuli [20]	2008		X		X		X		
Matthan [22]	2020		X	X		X	X		2 h insulin
Santiprabhob [23]	2018		X			X	X		
Arauz Boudreau [24]	2013	X			X			X	
Vilchis-Gil [25]	2018		X			X	X		
Kazankov [26]	2015			X	X		X		
Velazquez-Lopez [28]	2014		X	X		X	X		2 h insulin
Reinehr [29]	2010		X			X	X		
Reinehr [30]	2009		X	X					
Mayerhofer [33]	2020		X			X	X	X	
Rosenbaum [34]	2007		X			X			AIR, QUICKI
Pedrosa [35]	2011		X						
Gajewska [36]	2009		X						
Fernandez-Ruiz [37]	2021		X				X		
Moleres [38]	2013	X							
Marild [40]	2015		X			X	X	X	
Macknin [43]	2015		X			X		X	
Leal-Witt [44]	2018						X	X	
Reinehr [45]	2006		X			X	X		
Koncsos [46]	2011		X			X	X		
Fernandes [47]	2020		X			X	X		
Schwandt [48]	2011		X						
Reinehr [49]	2009		X			X	X		
Karampatsou [50]	2021		X			X	X	X	
Gajewska [51]	2018		X						
Pablos [54]	2018		X						
Izadpanah [55]	2012		X			X	X		QUICKI
Kahhan [57]	2021						X		

“X” indicates measurement of biomarker in selected study. Abbreviations: AIR—acute insulin response; Glc, plasma glucose; HbA1c—Hemoglobin A1c; HOMA-IR—Homeostatic Model Assessment for Insulin Resistance; QUICKI—quantitative insulin sensitivity check index.

**Table 6:** Biomarkers measured in papers measuring lipids.

Lead Author	Year Published	TGs	Total Cholesterol	HDL	LDL	Apo B	Apo A-I	Other
Huang [16]	2011	X	X	X				
Liao [17]	2021	X	X	X	X			
Cambuli [20]	2008	X	X	X	X			
Matthan [22]	2020	X	X	X	X			
Santiprabhob [23]	2018	X	X	X	X			
Arauz Boudreau [24]	2013	X	X	X	X			VLDL
Vilchis-Gil [25]	2018	X	X	X	X			
Kazankov [26]	2015	X	X	X	X			
Velazquez-Lopez [28]	2014	X	X	X	X			
Reinehr [30]	2009	X	X	X	X			
Rosenbaum [34]	2007	X	X	X	X			
Pedrosa [35]	2011	X	X	X	X	X	X	Apo A-I/B ratio
Gajewska [36]	2009	X	X	X	X			
Fernandez-Ruiz [37]	2021	X	X	X	X			
Moleres [38]	2013	X	X	X	X	X	X	LAP
Marild [40]	2015	X	X	X	X			
Macknin [43]	2015	X	X	X	X			
Leal-Witt [44]	2018	X	X	X	X			
Koncsos [46]	2011	X	X	X	X			
Fernandes [47]	2020	X	X	X	X			
Schwandt [48]	2011	X	X	X	X			LDL/HDL ratio, LDL/non-HDL-C ratio, TG/HDL ratio
Reinehr [49]	2009	X	X	X	X			
Karampatsou [50]	2021	X	X	X	X	X	X	Lp(a)
Gajewska [51]	2018	X	X	X	X			
Pablos [54]	2018	X	X					
Izadpanah [55]	2012	X	X	X	X			TC/HDL ratio, LDL/HDL ratio
Kahhan [57]	2021	X	X	X	X			

“X” indicates measurement of biomarker in selected study. Abbreviations: Apo, apolipoprotein; HDL, high-density lipoprotein; LDL; low-density lipoprotein; VLDL, very low-density lipoprotein; LAP, lipid accumulation profile; TG, triglyceride; Lp(a), lipoprotein a.

**Table 7:** Biomarkers measured in papers reporting any proinflammatory cytokines.

Lead Author	Year Published	TNF-α	IL-6	CRP	hsCRP	Other
Thomsen [19]	2021				X	
Matthan [22]	2020	X	X		X	IL-1
Santiprabhob [23]	2018		X		X	
Arauz Boudreau [24]	2013	X	X		X	
Kazankov [26]	2015	X	X		X	sCD163
Mayerhofer [33]	2020			X		
Rosenbaum [34]	2007	X	X	X		
Pedrosa [35]	2011			X		
Moleres [38]	2013			X		
Marild [40]	2015				X	
Macknin [43]	2015		X		X	MPO
Koncsos [46]	2011				X	
Karampatsou [50]	2021				X	
Hajihashemi [53]	2014				X	SAA
Izadpanah [55]	2012	X	X			IL-8, IL-1ra, IL-1β, IL-10, MCP-1
Kahhan [57]	2021	X	X		X	
Verbiest [42]	2021				X	

“X” indicates measurement of biomarker in selected study. Abbreviations: TNF-α; tumor necrosis factor alpha; IL-6, Interleukin 6; IL-1β, Interleukin-1 beta; CRP; C-reactive protein; hsCRP, high-sensitivity C-reactive protein; sCD163, Soluble CD163; MPO, myeloperoxidase; SAA, serum amyloid A; IL-8, Interleukin 8; IL-1ra, IL-1 receptor antagonist; IL-10, Interleukin 10; MCP-1, monocyte chemoattractant protein 1.

**Table 8:** Biomarkers measured in papers examining carotenoids.

Lead Author	Skin Carotenoids	Plasma Carotenoids	Lutein	Zeaxanthin	β-Cryptoxanthin	β-Carotene	Lycopene
Pratt [15]	X						
Matthan [22]		X	X	X	X	X	X
Hopkins [27]	X						
Juarez [31]	X						
Beccarelli [41]	X						

“X” indicates measurement of biomarker in selected study.

## Data Availability

All relevant data are included in the text of this manuscript.

## Supplementary Materials

The following supporting information can be downloaded at: https://www.mdpi.com/article/10.3390/nu16213584/s1, Table S1: Assessment of the quality of the studies included in the review.
